# Outcomes and Healthcare Resource Utilization in Patients with COVID-19 Treated with Nirmatrelvir–Ritonavir: Real-World Data Analysis

**DOI:** 10.3390/jcm13206091

**Published:** 2024-10-12

**Authors:** Clara Weil, Lilac Tene, Gabriel Chodick, Noga Fallach, Wajeeha Ansari, Tal Distelman-Menachem, Yasmin Maor

**Affiliations:** 1 Maccabi Institute for Research and Innovation, Maccabi Healthcare Services, Tel Aviv 6812509, Israelchodick@tauex.tau.ac.il (G.C.); fallach_n@mac.org.il (N.F.); 2 Pfizer Inc., New York, NY 10017, USA; wajeeha.ansari@pfizer.com; 3 Pfizer, Herzeliya 4672509, Israel; tal.distelman-menachem@pfizer.com; 4 Infectious Disease Unit, E. Wolfson Medical Center, Halochamim 62, Holon 5822012, Israel; 5 Faculty of Medical and Health Sciences, Tel Aviv University, Ramat Aviv, P.O. Box 39040, Tel Aviv 6997801, Israel

**Keywords:** SARS-CoV-2, nirmatrelvir–ritonavir, treatment, healthcare resource utilization, hospitalization, outcomes

## Abstract

**Background:** Nirmatrelvir–ritonavir was granted emergency use authorization in Israel in January 2022 to treat high-risk patients with mild-to-moderate COVID-19. The aim of the study was to assess the association between nirmatrelvir–ritonavir treatment and COVID-19-related hospitalization and healthcare resource utilization (HCRU) in a country with a high level of vaccinations compared to patients who were offered treatment and declined. **Methods:** The Maccabi Healthcare Services dataset was used to identify high-risk SARS-CoV-2-positive adults from January to February 2022 who received nirmatrelvir–ritonavir within 5 days of symptom onset (treatment group) or who were offered nirmatrelvir–ritonavir treatment and declined it (reference group). COVID-19-related hospitalizations and all-cause mortality and HCRU within 30 days were compared between treatment and reference groups using inverse probability of treatment weighting. **Results:** Treatment and reference groups included 3460 (median age, 68.4 years) and 1654 (70.2 years) patients, respectively. Patients with ≥1 dose of COVID-19 vaccine accounted for 89.5% (treatment group) and 72.1% (reference group) of the total. Treatment was associated with a lower risk of COVID-19-related hospitalization (adjusted OR, 0.59 [95% CI, 0.41,0.83]). Results were similar by age group (18–64/≥65 years) and among patients with/without vaccination in the prior 180 days. There were 11 (0.3%) versus 11 (0.7%) deaths in the treatment and reference groups, respectively. Treated patients had lower inpatient HCRU and greater less intensive outpatient HCRU (e.g., telemedicine and emergency room visits). **Conclusions:** Nirmatrelvir–ritonavir treatment was associated with a reduced risk of COVID-19-related hospitalization and a shift to less intensive outpatient HCRU. Comparison with a reference group of nirmatrelvir–ritonavir-eligible patients who declined treatment enabled an unbiased outcome assessment. Real-world data gathered during the Omicron BA.1 variant wave of COVID-19 in Israel support the continued use of nirmatrelvir–ritonavir for high-risk adults of all ages, regardless of previous vaccinations.

## 1. Introduction

Coronavirus disease 2019 (COVID-19) is a global health threat, commonly spread through respiratory droplets and aerosol transmission and whose causative agent, severe acute respiratory syndrome coronavirus-2 (SARS-CoV-2), is an enveloped positive single-stranded RNA virus, responsible for pulmonary and also extrapulmonary organ infection [1]. The COVID-19 pandemic started in a seafood market in Wuhan, China, in early December 2019, caused more than 7 million deaths worldwide, and continues to threaten global health [2]. Infection with SARS-CoV-2, the consensus source of the pandemic, may lead to severe disease, including hospitalizations, intensive care unit (ICU) stays, and invasive mechanical ventilation. Risk factors for severe disease, complications, and death include an age of ≥65 years and underlying medical conditions such as diabetes, cardiovascular disease, respiratory disease, and immunosuppression [3].

Before the introduction of targeted oral antivirals, treatment options were limited for patients with COVID-19 early in the course of the disease and were primarily administered in a hospital setting to patients who had progressed to severe disease. The availability of oral drugs administered early in the disease course reduced the need for hospitalization and shifted treatment into community settings. One of the leading interventions is nirmatrelvir–ritonavir, an orally administered antiviral agent targeting the SARS-CoV-2 main protease enzyme essential for viral replication [4,5,6]. Nirmatrelvir, the active antiviral against SARS-CoV-2 is administered together with ritonavir, which boosts the plasma concentration of nirmatrelvir to efficacious levels. EPIC-HR (Evaluation of Protease Inhibition for COVID-19 in High-Risk Patients) was a randomized, double-blind study of 2246 non-hospitalized, symptomatic, unvaccinated adult patients with COVID-19 who were at high risk of progressing to severe illness. Compared to the placebo, nirmatrelvir reduced the combined outcome of hospitalization and death by 89%, eliminated death as an outcome, and reduced viral load by 0.87 log_10_ copies per milliliter after 5 days of treatment. This critical result prompted the Food and Drug Administration (FDA) to issue an Emergency Use Authorization. The drug became the most widely prescribed antiviral for SARS-CoV-2 in the United States, likely preventing thousands of hospitalizations and many deaths [7]. Nirmatrelvir–ritonavir also demonstrated a tolerable safety profile [6,7]. Based on these results, on 22 December 2021, the US Food and Drug Administration issued an Emergency Use Authorization (EUA) for nirmatrelvir–ritonavir. A similar EUA was issued in Israel by the Ministry of Health (MoH) on 26 December 2021, and treatment became available to eligible high-risk patients in January 2022, a time of widespread infection with the SARS-CoV-2 Omicron BA.1 variant [8,9,10]. Ritonavir-boosted nirmatrelvir was recently licensed by the FDA based on its continued effectiveness and safety and has outperformed other antivirals in terms of preventing hospitalization and viral load reduction [11].

Several published real-world studies from Hong Kong, Israel, and the United States have demonstrated the effectiveness of nirmatrelvir–ritonavir for preventing severe outcomes (typically hospitalization and/or death), with some showing different effectiveness in elderly versus younger populations [12,13,14,15,16,17,18,19]. This study aimed to assess the association between nirmatrelvir–ritonavir treatment and COVID-19-related hospitalization in a country with a high level of vaccination using a unique comparator group of patients who were offered nirmatrelvir–ritonavir treatment in a large health plan in Israel and actively declined it. In addition, we aimed to assess the effect of treatment with nirmatrelvir–ritonavir on healthcare resource utilization (HCRU) in inpatient and outpatient settings.

## 2. Materials and Methods

### 2.1. Data Source

A retrospective cohort study was performed using electronic healthcare data from Maccabi Healthcare Services (MHS), a large health plan serving 25% of the Israeli population. MHS covers, by law, all hospitalizations in general hospitals outside the MHS network, as well as visits to emergency rooms (ERs) in out-of-network hospitals (including cases in which a co-pay is required). The MHS database contains more than two decades of longitudinal data on >2.6 million members, including diagnosis, pharmacy, and laboratory data (including SARS-CoV-2 test results from MHS and from all other authorized laboratories in Israel via the Israeli Ministry of Health). The study was approved by the MHS Institutional Review Board (approval number MHS-0030-22). Informed consent was waived because of the retrospective nature of the study. Data were accessed and downloaded between 1 April 2022 and 14 December 2022. All data were fully anonymized.

### 2.2. Study Population

During the study period from January to February 2022, nirmatrelvir–ritonavir was the preferred antiviral treatment for COVID-19 for MHS members. Patients’ eligibility for receiving nirmatrelvir–ritonavir was assessed by a multidisciplinary team from the MHS COVID-19 Antiviral Treatment Center. The team was responsible for (1) identifying and assessing patient eligibility for COVID-19 antiviral treatment (nirmatrelvir–ritonavir or molnupiravir); (2) contacting patients, reviewing their eligibility, and affirming informed consent for the chosen treatment; and (3) delivering treatment to patients’ homes. Each day, the Treatment Center physicians received a list of patients newly diagnosed with SARS-CoV-2 (through formal institutional testing via a reverse transcription polymerase chain reaction [RT-PCR] or antigen test) with known risk factors associated with progression to severe COVID-19. Physicians reviewed patients’ electronic health records (EHRs) to determine eligibility for treatment based on age, comorbidities, and concomitant medication use. Physicians then contacted eligible patients by phone and offered COVID-19 antiviral treatment. Although data on symptom onset were not available in the EHRs, no patient received treatment with nirmatrelvir–ritonavir unless it could be given within five days of symptom onset as ascertained through the phone call when treatment options were being discussed. For patients who were not eligible for nirmatrelvir–ritonavir because of drug–drug interactions or renal insufficiency, molnupiravir was offered. The patient’s choice to accept or decline this treatment (status) was recorded. For patients who opted to receive treatment, the medication was transported to the patient’s home within several hours and the first dispense date was used to define the study index date (treatment group). For patients who declined and were not provided treatment (reference group), the status date was used to define the study index date.

Patients were eligible for inclusion in this analysis if they had a record of being offered nirmatrelvir–ritonavir treatment (as documented by the COVID-19 Antiviral Treatment Center) with a positive SARS-CoV-2 result (via RT-PCR or a formal antigen test); an index date occurring through 28 February 2022; and continuous enrollment in MHS for ≥12 months pre-index. Eligibility for nirmatrelvir–ritonavir was defined by the MHS COVID-19 Antiviral Treatment Center, as defined above. To ensure that there was documented evidence of high-risk eligibility in the EHRs, we excluded patients who were 50–69 years old with a risk score < 2 points and patients 18–49 years old with <4 points, based on a risk score that accounts for comorbidities (such as cardiovascular disease, immunosuppression), hospitalization in the past three years, and COVID-19 vaccination history (risk score definitions are provided in the Supplementary Materials, Table S1). Patients were also excluded from the study if they had a positive SARS-CoV-2 test result in the six months before the current positive test date, were hospitalized on/after their positive test date or index date before receiving antiviral treatment or were given molnupiravir.

### 2.3. Study Outcomes and Variables

The primary outcomes were COVID-19-related hospitalization (in a COVID-19 ward or a hospitalization recorded by the Israeli MoH as being due to COVID-19 [20]) and all-cause HCRU within 30 days after the index date. HCRU data included all-cause hospitalizations (in any ward, including the ICU, with or without evidence of being COVID-19-related) and visits to any of the following: the ER, after-hours urgent care, a primary care physician, or a specialist physician. Telemedicine visits were reported separately. As an exploratory outcome, total costs per patient associated with all-cause HCRU and pharmacy expenses over the 30-day follow-up period were calculated using unit costs from the Israel MoH price list [20]. For the exploratory outcome of all-cause mortality, the death date was obtained from Israel’s National Insurance Institute [21].

Patients’ sociodemographic characteristics were described at the index date, including sex, age, area of residence, residential-based population subgroup (ultra-orthodox Jewish, Arab, other), and residential socioeconomic status (SES). SES was based on a score ranked from 1 (lowest) to 10 (highest), derived by the Israel Central Bureau of Statistics [22,23], Body mass index in kg/m [2] was based on the last measure up to five years pre-index and classified as underweight (<18.5), normal (18.5–24.9), overweight (25.0–29.9), obese (30.0–39.9), and morbid obesity (≥40).

Comorbidities were recorded before/at index and identified by existing MHS disease registries [24,25,26,27] when available (diabetes, cardiovascular disease, chronic kidney disease, hypertension, chronic obstructive pulmonary disease, and immunosuppression, including the National Cancer Registry) or by the International Classification of Diseases, Ninth Revision, Clinical Modification (ICD-9-CM) diagnosis codes (history of chronic liver disease and diseases of the nervous system in the prior 12 months). Smoking status (ever vs. never) was obtained from physician reports.

COVID-19 vaccinations and prior SARS-CoV-2 infections (ever and in the prior 180 days) were described according to (1) the number of patients receiving at least one vaccination dose, (2) the number of vaccine doses received, and (3) a receipt of ≥1 vaccination dose in the prior 180 days. Data were also obtained regarding the most recent symptoms using the COVID-19 Symptoms Questionnaire, which was completed during a physician visit up to 5 days before or at the index date (and no earlier than the SARS-CoV-2-positive test date), when available. This questionnaire assessed the presence of the following symptoms: fever, difficulty breathing, cough, loss of taste or smell, weakness, muscle pain, or diarrhea.

### 2.4. Statistical Analyses

Descriptive statistics are presented as n (%), mean (SD), or median (interquartile range [IQR]), as appropriate. Comparisons of baseline characteristics across groups were performed using the chi-square test, Student’s *t* test, or the Wilcoxon rank-sum test, depending on the distribution of the data. The standardized mean difference was reported. A propensity score-based methodology was used to adjust for potential measured selection and confounder biases. Propensity scores representing the probability of treatment were calculated for each patient with a logistic regression model including baseline patient characteristics and the calendar week of the index date. Inverse probability treatment weighting (IPTW) based on the propensity score was used in all analyses to balance the groups regarding baseline characteristics. Standard diagnostics for propensity scores were performed, exploring potential collinearity and tests for balance.

COVID-19-related hospitalization and all-cause HCRU were described for the treatment group, before and after IPTW. Odds ratios and 95% CIs from logistic regressions were reported per healthcare resource (i.e., odds of ≥1 hospitalization/visit/unit of resource within 30 days post-index) for treatment versus reference groups. Mean total healthcare costs per patient during the 30-day period (in USD purchasing power parities [26]) were assessed using generalized linear models with log link and gamma distribution. Where more than 5% contained zero values, a two-part model was implemented. IPTW was implemented to all multivariable models; results were presented before and after weighing. If any adjustment variable had a standardized mean difference (SMD) greater than 0.1 after IPTW, that variable was included as an additional term in the model. All SMDs with IPTW, however, were <0.1, so no further adjustment was made. In an exploratory analysis, all-cause mortality was described and IPTW-adjusted hazard ratios (HRs) were obtained from Cox regression.

Analyses were repeated for the 18–64- and ≥65-year-old age groups. Separate propensity scores and IPTW were computed for each age group. For the primary outcome (COVID-19 hospitalization), results were also stratified by those with/without vaccination in the prior 180 days.

Alpha was set to 0.05 for all comparisons in this study. Data analyses were performed using IBM-SPSS v.28 and R statistical software v.4.0.2.

## 3. Results

**Figure 1:**
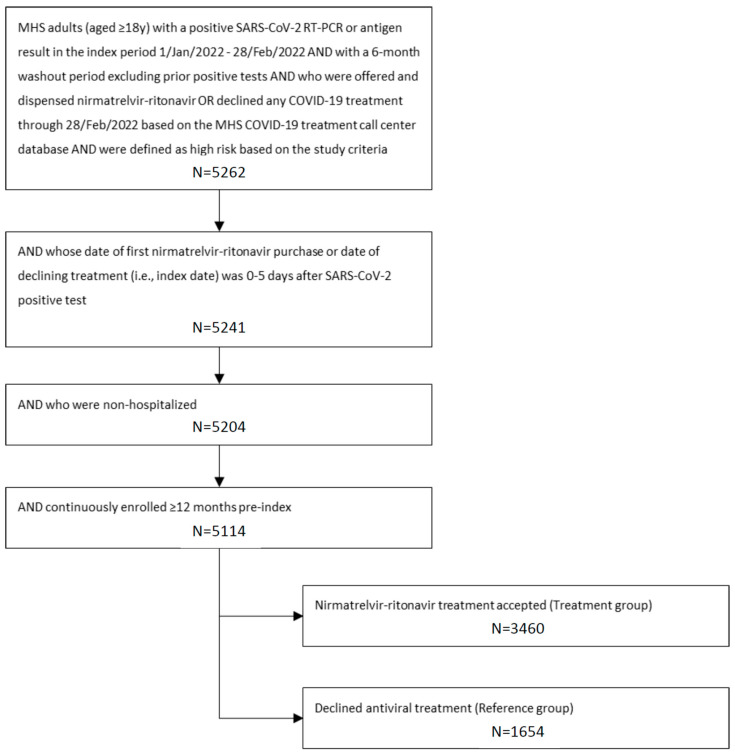
Study flowchart. MHS = Maccabi Healthcare Services.

The study population included 3460 patients in the treatment group and 1654 patients in the reference group (Figure 1). The median (IQR) age in the treatment and reference groups was 68.4 (59.8, 75.5) and 70.2 (60.6, 77.3), respectively (Table 1). Females comprised 49.6% and 49.3% in the treatment and reference groups, respectively. Patients in both groups had a median of 1 day between their SARS-CoV-2-positive test and index date. Treated patients (vs. reference) were more likely to be vaccinated with ≥1 dose (89.5% vs. 72.1%) and with a higher number of doses; patients vaccinated in the prior 180 days accounted for 72.4% vs. 51.4%, respectively. The baseline prevalence of immunosuppression was similar in the treatment (26.7%) and reference (28.1%) groups. Differences in key sociodemographic and clinical characteristics between the two groups were well balanced after IPTW.

A total of 32 (0.9%) versus 34 (2.1%) patients in the treatment vs. reference groups, respectively, had a COVID-19-related hospitalization (median length of stay: 5.0 vs. 5.5 days). After adjustment, treatment use was associated with an overall lower risk of COVID-19-related hospitalization (adjusted OR, 0.59 [95% CI, 0.41, 0.83]). Treatment use was associated with an overall lower risk of COVID-19-related hospitalization also in stratified analyses by age group and receipt of COVID-19 vaccination in the prior 180 days (Table 2). In a subgroup-adjusted analysis, treatment was associated with significantly less COVID-19-related hospitalizations in women but not in men (see Supplementary Materials for analyses for hospitalization by gender).

Among patients with COVID-related hospitalizations, eight patients in each study group were admitted to the ICU (three additional patients in each group were admitted to the ICU, but the indication was not COVID-19-related).

Patients in the treatment (vs. reference) group had numerically lower rates of all-cause hospitalization (2.8% vs. 3.9%, respectively). Death within 30 days post-index occurred in 11 (0.3%) and 11 (0.7%) patients (adjusted HR, 0.89 [95% CI, 0.50, 1.61]; see Supplementary Materials Table S2 for age-stratified analyses).

Patients in the treatment versus reference group had lower inpatient HCRU and greater use of less intensive outpatient HCRU, such as ER visits (in the older population), urgent care services (in the younger population), and telemedicine visits (Table 3 and Supplementary Materials, Tables S3–S5 for age-stratified results). In a subgroup-adjusted analysis, treatment was associated with significantly less HCRU in women but not in men (see Supplementary Materials Table S6 for analyses for hospitalization by gender).

Estimated mean total costs per person associated with all-cause HCRU within 30 days post-index were similar in both groups (adjusted ratio of means, 1.10 [95% CI, 0.96, 1.26]) and in age-stratified analyses.

## 4. Discussion

In this real-world retrospective study of patients with COVID-19 during the Omicron BA.1 wave in Israel, patients treated with nirmatrelvir–ritonavir had a substantially lower risk of COVID-19-related hospitalization within 30 days compared with those who declined the treatment. This difference was seen across patient subgroups by age (18–64 and ≥65 years) and among those with/without COVID-19 vaccination in the prior 180 days. Treatment was also associated with an overall shift to less intensive HCRU. However, there were no significant differences in the estimated mean total costs per person associated with all-cause HCRU. All-cause mortality was relatively low in both groups (treatment, 0.3%; reference, 0.7%).

The treatment group shifted to the use of the outpatient resources of the ER (in the older population), urgent care services (younger population), and telemedicine visits, and the reference group had higher inpatient HCRU, where treatment and diagnostic examinations that otherwise would be carried out in the outpatient setting could have occurred. This shift may suggest differences in healthcare-seeking behavior, severity of comorbidities, and/or the severity of COVID-19.

Our findings are consistent with emerging real-world data describing the effectiveness of nirmatrelvir–ritonavir in reducing COVID-19-related hospitalizations. With real-world data from a different Israeli health plan, Arbel et al. [12] found that nirmatrelvir–ritonavir was associated with decreased hospitalization in patients ≥65 years old, similar to results in this study. However, they did not identify a beneficial effect of nirmatrelvir–ritonavir in younger adults. This difference may be due to methodological differences in the age of the younger cohort (40–64 years in Arbel et al. vs. 18–64 years in this study), risk factors and treatment eligibility criteria, and in the selection of their reference group.

This study is unique in that all reference group patients were offered nirmatrelvir–ritonavir treatment but opted against it. Comparator groups in other real-world evidence studies [20,22,24,25] did not specify the reasons patients were not treated with nirmatrelvir–ritonavir, which may have included active refusal (regardless of the reason for refusal, as in our reference group), but could also have included unawareness of COVID-19 treatments, unavailability of nirmatrelvir–ritonavir, lack of indication for any treatment (e.g., younger patients without risk factors for COVID-19 deterioration), contraindication for treatment (e.g., renal failure or drug–drug interactions), and differences in health-seeking behaviors that may alter patient acceptance of drugs administered through an EUA program [28,29]. By selecting a reference group that went through the same process for assessing treatment eligibility as those who were ultimately treated, we attempted to reduce the potential for selection bias.

In a subgroup analysis, the adjustment treatment was associated with significantly less COVID-19-related hospitalizations and HCRU in women but not in men. This was probably related to the small sample size after dividing into subgroups rather than a true difference in the effect of treatment related to gender, as this difference was not found in other publications [30,31]. The results of this study should be interpreted within certain limitations in addition to the inherent limitations of real-world data. This study captures the early use of nirmatrelvir–ritonavir in the first weeks following the EUA in Israel during a pandemic and public health emergency, resulting in unique environmental and other related challenges possibly affecting the evaluation of the real-world use of nirmatrelvir–ritonavir. The MHS COVID-19 Antiviral Treatment Center assessed patient eligibility for nirmatrelvir–ritonavir treatment but the precise reason(s) that a given patient was deemed eligible/ineligible for treatment was not specified. To address the problem of a limited stock of nirmatrelvir–ritonavir, criteria to designate eligibility were adjusted daily according to drug availability. Therefore, the study group may not have been uniform over time in terms of risk criteria, and this could have affected the group comparability.

Although eligibility was verified for each patient through a phone call with a physician, including ensuring that treatment would be given within 5 days of symptom onset, the actual date of symptom onset was not recorded. Additionally, the COVID-19 Symptoms Questionnaire describes the presence, but not severity, of COVID-19 symptoms, so differences in symptom severity may exist between the two groups. Another limitation is that patients were potentially offered treatment at differing time points across their disease course or symptom progression, which potentially influenced both their decision to accept treatment and the outcome. It is possible that patients who had already started to improve were more likely to decline treatment. Both limitations in symptom data could favor the reference group. It was not possible to control for certain intangible confounders, such as health-seeking behavior.

Several studies have discussed drug–drug interactions (DDIs) related to the treatment with nirmatrelvir–ritonavir [32] and appropriate dosing regarding renal function and DDIs [33]. Unfortunately, we did not have these data available to us, so we cannot rule out that some of the prescriptions were inappropriate regarding DDIs and dose adjustment.

HCRU data, other than hospitalization data, could not be specifically attributed to COVID-19, and thus may include costs and utilization related to other conditions. Hospitalization costs were calculated using the Israeli MoH’s standard per diem unit costs at public hospitals. This methodology ensures that calculations of cost are consistent with MoH standard service tariff rates by length of stay and by site of service but can obscure patient-level differences in intensity of care. The per diem unit costs also may not be generalizable to other countries and care settings (e.g., the per diem unit costs are much lower than costs estimated in the United States).

To conclude, our data showed that nirmatrelvir–ritonavir was associated with a reduced risk of COVID-19-related hospitalizations and a shift to less intensive outpatient HCRU during Omicron BA.1 predominance in a highly vaccinated, high-risk cohort, after weighting to ensure comparability between study groups. Differences favoring nirmatrelvir–ritonavir treatment remained after subgroup analyses and were observed in vaccinated and unvaccinated patients 18 to 64 and ≥65 years old. These results support the treatment of adult high-risk patients in all age groups, regardless of previous vaccination.

## Figures and Tables

**Table 1:** Demographics and clinical characteristics at index date, before and after IPTW.

Characteristic	Unweighted			IPTW ^c^		
	Reference Group ^a^ n = 1654	Treatment Group ^a^ n = 3460	SMD	Reference Group ^b^	Treatment Group ^b^	SMD
Age, y			0.1			0.01
Median (IQR)	70.2 (60.6, 77.3)	68.4 (59.8, 75.5)		70.1 (60.4, 76.8)	68.9 (60.3, 76.0)	
≥65 ^c^	1044 (63.1)	2091 (60.4)	0.06	62.5	61.8	0.01
Sex			0.01			0.02
Female	815 (49.3)	1716 (49.6)		50.5	49.7	
Socioeconomic status, residential			0.31			0.02
Low	341 (20.6)	480 (13.9)		16.8	16.1	
Medium	642 (38.8)	1056 (30.5)		33.9	33.8	
High	666 (40.3)	1918 (55.4)		49.1	49.8	
Missing	5 (0.3)	6 (0.2)		0.3	0.2	
Smoking status			0.09			0.02
Never	1473 (89.1)	3156 (91.2)		91.1	90.7	
Ever	177 (10.7)	288 (8.3)		8.5	8.9	
Missing	4 (0.2)	16 (0.5)		0.4	0.4	
BMI category, kg/m^2^			0.08			0.02
Normal, 18.5–24.9	351 (21.2)	714 (20.6)		20.5	20.8	
Underweight, <18.5	9 (0.5)	23 (0.7)		0.8	0.6	
Overweight, 25.0–29.9	584 (35.3)	1286 (37.2)		36.5	36.3	
Obese I, 30.0–39.9	576 (34.8)	1169 (33.8)		34.6	34.6	
Obese II, 40.0+	58 (3.5)	147 (4.2)		3.8	3.8	
Missing	76 (4.6)	121 (3.5)		3.8	3.8	
Diabetes	624 (37.7)	1233 (35.6)	0.04	36.2	36.5	–0.01
Cardiovascular disease	573 (34.6)	1093 (31.6)	0.06	32.5	32.5	0
Hypertension	1092 (66.0)	2068 (59.8)	0.13	63.3	62.3	0.02
Chronic kidney disease	718 (43.4)	1357 (39.2)	0.09	41.0	40.8	0
Liver disease	423 (25.6)	923 (26.7)	–0.03	25.6	26.1	–0.01
Diseases of the nervous system in prior 12 mo	917 (55.4)	2010 (58.1)	–0.05	58.5	57.5	0.02
Chronic obstructive pulmonary disease	136 (8.2)	300 (8.7)	–0.02	8.8	8.5	0.01
Cancer, active or treated in prior 5 y	208 (12.6)	511 (14.8)	–0.06	14.5	14.2	0.01
Immunosuppression	465 (28.1)	924 (26.7)	0.03	27.5	27.0	0.01
Hospitalization in prior 180 d	182 (11.0)	353 (10.2)	0.03	10.5	10.5	0
Risk score category ^c^			0.28			0.04
2	186 (11.2)	685 (19.8)		14.9	17.2	
3	248 (15.0)	645 (18.6)		18.6	16.7	
4+	1220 (73.8)	2130 (61.6)		66.5	66.0	
Risk score = 4+	1220 (73.8)	2130 (61.6)	0.26	66.5	66.0	0.01
Symptoms questionnaire, between SARS-CoV-2-positive test and index date			0.29			0
None reported	159 (9.6)	97 (2.8)		5.3	5.3	
1+ reported	833 (50.4)	1760 (50.9)		50.7	50.6	
Missing	662 (40.0)	1603 (46.3)		44.0	44.1	
Days since SARS-CoV-2-positive test ≥ 3	122 (7.4)	157 (4.5)	0.12	5.5	5.3	0.01
COVID-19 vaccination status, doses			–0.45			0
0	462 (27.9)	365 (10.5)		16.9	17.2	
1	30 (1.8)	61 (1.8)		2.1	1.9	
2	84 (5.1)	183 (5.3)		5.7	5.3	
3	703 (42.5)	1716 (49.6)		46.0	46.5	
4	375 (22.7)	1135 (32.8)		29.3	29.1	
COVID-19 vaccination status in prior 180 d	850 (51.4)	2504 (72.4)	–0.44	64.6	64.8	0
Prior SARS-CoV-2 infection	99 (6.0)	128 (3.7)	0.11	4.5	4.7	–0.01

BMI = body mass index; IPTW = inverse probability of treatment weighting; IQR = interquartile range; SARS-CoV-2 = severe acute respiratory syndrome *coronavirus 2*; SMD = standardized mean difference. ^a^ All data are n (%) unless otherwise stated. ^b^ All data are percentages unless otherwise stated. ^c^ IPTW was used to adjust for differences in patient characteristics associated with the probability of receiving treatment vs. declining treatment. The propensity score model included all characteristics in the table above, as well as “calendar week of index date”. Age was included in the propensity score model as a continuous variable (rather than “age group”), and risk score was included as a binary variable (“risk score = 4+” rather than “risk score category”); the SMD associated with these variables was used to assess covariate balance between the two groups.

**Table 2:** COVID-19-related hospitalization within 30 days post-index date, before and after IPTW, overall, and by age group or vaccination status.

		COVID-19-Related Hospitalization, 30 d Post-Index: Treatment (n = 3460) vs. Reference (n = 1650) Group			
Age Group at Index, y	COVID-19 Vaccination Status at Index	Unweighted		IPTW ^a^	
		OR	95% CI	OR	95% CI
Overall	Overall	**0.44**	**0.27, 0.72**	**0.59**	**0.41, 0.83**
	Not vaccinated in prior 180 days ^b^	0.53	0.26, 1.03	**0.59**	**0.37, 0.93**
	Vaccinated in prior 180 days	0.51	0.24, 1.08	**0.59**	**0.35, 0.97**
18–64	Overall	0.52	0.23, 1.20	**0.46**	**0.26, 0.78**
	Not vaccinated in prior 180 days ^b^	0.65	0.21, 1.97	**0.47**	**0.21, 0.98**
	Vaccinated in prior 180 days	0.47	0.14, 1.79	**0.45**	**0.20, 0.97**
≥65	Overall	**0.41**	**0.22, 0.75**	**0.59**	**0.38, 0.92**
	Not vaccinated in prior 180 days ^b^	0.49	0.19, 1.13	0.59	0.32, 1.05
	Vaccinated in prior 180 days	0.52	0.21, 1.34	0.61	0.30, 1.17

COVID-19 = coronavirus disease 2019; IPTW = inverse probability of treatment weighting; OR = odds ratio. ^a^ IPTW was used to adjust for differences in patient characteristics associated with the probability of receiving treatment versus declining treatment. The propensity score model included all characteristics in the table above, as well as “calendar week of index date”. Age was included in the propensity score model as a continuous variable (rather than “age group”), and risk score was included as a binary variable (“risk score = 4+” rather than “risk score category”); the SMD associated with these variables was used to assess covariate balance between the two groups. ^b^ Includes patients who were never vaccinated and patients who were vaccinated >180 days pre-index. Marking by bold indicates statistical significance.

**Table 3:** HCRU within 30 days post-index date, before and after IPTW, and overall.

	Unweighted				IPTW ^c^			
Characteristic	Reference Group n = 1654 ^a^	Treatment Group n = 3460 ^a^	SMD	OR (95% CI)	Reference Group ^b^	Treatment Group ^b^	SMD	OR (95% CI)
PCP, ≥1 visit	1614 (97.6)	3394 (98.1)	−0.04	1.27 (0.85, 1.89)	97.9	98.2	−0.02	1.16 (0.88, 1.54)
Specialists, ≥1 visit	544 (32.9)	1263 (36.5)	−0.08	**1.17 (1.04, 1.33)**	35.8	35.6	0	0.99 (0.91, 1.08)
Telemedicine, ≥1 visit	804 (48.6)	1931 (55.8)	−0.14	**1.34 (1.19, 1.50)**	50.3	54.0	−0.07	**1.16 (1.07, 1.25)**
After-hours urgent care, ≥1 visit	6 (0.4)	20 (0.6)	−0.03	1.6 (0.68, 4.37)	0.3	0.5	−0.03	1.73 (0.94, 3.30)
ER, ≥1 visit	92 (5.6)	203 (5.9)	−0.01	1.06 (0.82, 1.37)	5.10	6.10	−0.04	**1.2 (1.02, 1.43)**
Hospitalization (all-cause), ≥1 admission	65 (3.9)	97 (2.8)	0.06	**0.71 (0.51, 0.97)**	3.4	3.1	0.02	0.92 (0.73, 1.14)
Hospitalization (all-cause), LOS			0.23				0.3	
n	65	97			168	159		
Median (IQR)	4.0 (2.0, 7.0)	3.0 (2.0, 6.0)			4.0 (2.0, 7.0)	3.0 (2.0, 5.4)		
Hospitalization (all-cause) in ICU, ≥ admission	10 (0.6)	10 (0.3)	0.05	0.48 (0.20, 1.16)	0.5	0.3	0.04	0.58 (0.31, 1.06)
Hospitalization (all-cause) in ICU, LOS			1.2				1.1	
n	10	10			27	16		
Median (IQR)	15.5 (3.5, 23.5)	2.5 (1.3, 8.0)			11.9 (2.3, 22.7)	3.2 (1.1, 8.0)		
Hospitalization, COVID-19-related, ≥1 admission	34 (2.1)	32 (0.9)	0.09	**0.44 (0.27, 0.72)**	1.7	1.0	0.06	**0.59 (0.41, 0.83)**
Hospitalization, COVID-19-related, LOS			0.37				0.56	
n	34	32			87	53		
Median (IQR)	5.5 (3.0, 11.0)	5.0 (3.0, 7.2)			6.0 (3.0, 11.9)	5.0 (2.4, 7.0)		
Hospitalization, COVID-19-related in ICU, LOS			1.5				1.5	
n	8	8			22	14		
Median (IQR)	19.0 (12.5, 24.0)	5.5 (1.8, 8.2)			15.5 (6.2, 23.9)	5.0 (1.5, 8.0)		
Maximum level of care			0.1				0.07	
None	29 (1.8)	46 (1.3)			1.5	1.3		
Telemedicine	91 (5.5)	203 (5.9)			5.0	5.7		
Outpatient	1431 (86.5)	3000 (86.7)			87.7	86.6		
ER	38 (2.3)	114 (3.3)			2.4	3.4		
Inpatient	55 (3.3)	87 (2.5)			2.8	2.8		
ICU	10 (0.6)	10 (0.3)			0.5	0.3		

COVID-19 = coronavirus disease 2019; ER = emergency room; HCRU = healthcare resource use; ICU = intensive care unit; IPTW = inverse probability of treatment weighting; IQR = interquartile range; LOS = length of stay; OR = odds ratio; PCP = primary care physician; SMD = standardized mean difference. Underline: SMD >10%. ^a^ All data are n (%) unless otherwise stated. ^b^ All data are percentages unless otherwise stated. ^c^ IPTW was used to adjust for differences in patient characteristics associated with the probability of receiving treatment versus declining treatment. The propensity score model included all characteristics in the table above, as well as “calendar week of index date”. Age was included in the propensity score model as a continuous variable (rather than “age group”), and risk score was included as a binary variable (“risk score = 4+” rather than “risk score category”); the SMD associated with these variables was used to assess covariate balance between the two groups. Marking by bold indicates statistical significance.

## Data Availability

Data used for this study are available from the study authors.

## Supplementary Materials

The following supporting information can be downloaded at: https://www.mdpi.com/article/10.3390/jcm13206091/s1, Supplementary Methods; results, Table S1: Risk points used to define the study population of antiviral treatment-eligible patients at high risk for deterioration of COVID-19; Table S2: Demographics and clinical characteristics at Index Date, Before and After IPTW, Overall, and by Age Group (18–64 years, ≥65 years); Table S3: HCRU Within 30 Days Post-Index Date, Before and After IPTW, Overall and by Age Group (18–64 Years, ≥65 Years); Table S4: Odds Ratios of HCRU Within 30 Days Post-Index Date, Before and After IPTW, Overall and by Age Group; Table S5: All-Cause Mortality, Before and After IPTW, Overall and by Age Group; Table S6: Odds Ratios of Hospitalization and HCRU Within 30 Days Post-Index Date, Before and After IPTW, for females and males.

## Abbreviations

COVID-19—coronavirus disease 2019; SARS-CoV-2—severe acute respiratory virus *coronavirus 2*; HCRU—healthcare resource utilization; OR—odds ratio; CI—confidence interval; FDA—Food and Drug Administration; ICU—intensive care unit; EPIC-HR—Evaluation of Protease Inhibition for COVID-19 in High-Risk Patients; US—United States; EUA—Emergency Use Authorization; MoH—Ministry of Health (MoH); MHS—Maccabi Healthcare Services; ER—emergency room; RT-PCR—reverse transcription polymerase chain reaction; EHRs—electronic health records; SES—socioeconomic status; ICD-9-CM—International Classification of Diseases, Ninth Revision, Clinical Modification; SD—standard deviation; IQR— interquartile range; IPTW—inverse probability treatment weighting; USD—United States dollar; SMD—standardized mean difference; BMI—body mass index; PCP—primary care physician; LOS—length of stay; DDI—drug–drug interaction.
