# Supplementary material for: Outcomes and Healthcare Resource Utilization in Patients with COVID-19 Treated with Nirmatrelvir–Ritonavir: Real-World Data Analysis
Source: J Clin Med. 2024 Oct 12;13(20):6091. doi: 10.3390/jcm13206091 (PMC11508699; doi:10.3390/jcm13206091)
Supplement: Supplementary file 1 [file jcm-13-06091-s001.zip › jcm-3221911-supplementary.pdf]

Supplementary Materials

Supplementary Methods

The residential SES measure was originally derived by the Israel Central Bureau of Statistics using national census data and augmented by POINTS location profiling Ltd., using aggregated data on housing prices, motorization level, education, employment, and financial resources.[20] Socioeconomic status was categorized as low (1-4), medium (5-6) and high (7-10).

Table S1. Risk points used to define the study population of antiviral treatment-eligible patients at high risk for deterioration of COVID-19

Risk factor	Definition	Risk points
Age[a]	NA	NA
Obesity	Body mass index ≥ 30 kg/m^2, according to the most recent measure in the prior 5 y	1
Diabetes	Existing MHS registry[22]	1
Cardiovascular disease	Existing MHS registry[23]	1
Chronic kidney disease	Existing MHS registry[24]	1
Chronic liver disease	ICD-9 codes 570-573	1
Neurologic disease	ICD-9 codes 320-389 (≥2 on separate dates)	1
Active malignancy or malignancy treated during the past 5 years	Existing registry; MHS pharmacy data	1
Immunosuppression	Existing MHS registry (including solid organ and bone marrow transplant, asplenia, recently dispensed oncologic and non-oncologic immunosuppressive treatments, advanced chronic kidney disease and other diagnosed immunosuppression)	1
Pregnancy	Existing MHS registry	1

Recent hospitalization	Hospitalization in the past 3 y (not including delivery/birth)	1 per admission
Lung disease or smoking history	Existing MHS registry for COPD; ICD-9 codes for asthma (493), bronchiectasis (494), interstitial lung disease (516.3), pulmonary embolism (515.1), pulmonary hypertension (416.0), cystic fibrosis (277.0) and lung transplant (V42.6); history of tobacco use disorder and physician reports of smoking	1
COVID-19 vaccination and prior SARS-CoV-2 positive test results	Unvaccinated and never infected	3
	1 dose and never infected	2
	2 doses and >6 mo since last dose	2
	3 doses and >6 mo since last dose	1
	Unvaccinated and infected >3 mo prior	1

COPD = chronic obstructive pulmonary disease; ICD-9 = *International Classification of Diseases, 9th Revision;* MHS = Maccabi Healthcare Services; NA = not applicable.
[a]Age group was not included in the risk score but was used to define risk levels, with high risk defined as (i) age ≥70 years, (ii) age 50–69 years with a risk score ≥2 points, and (iii) age <50 years with a risk score ≥4 points. This high-risk definition was applied to patients who were deemed eligible for antiviral treatment in clinical practice by physicians in the MHS COVID-19 antiviral treatment center through February 2022.

Supplementary Results

Table S2. Demographics and clinical characteristics at Index Date, Before and After IPTW, Overall, and by Age Group (18–64 years, ≥65 years)

(A) Patient Characteristics at Index Date, Overall

	**Unweighted**			**IPTW**[c]		
**Characteristic**	**Reference group n = 1,654**[a]	**Treatment group n = 3,460**[a]	**SMD**	**Reference group**[b]	**Treatment group**[b]	**SMD**
Age, y			0.1			0.01
Median (IQR)	70.2 (60.6, 77.3)	68.4 (59.8, 75.5)		70.1 (60.4, 76.8)	68.9 (60.3, 76.0)	
≥65	1,044 (63.1)	2,091 (60.4)	0.06	62.5	61.8	0.01

Characteristic	Unweighted Reference group n = 1,654[a]	Unweighted Treatment group n = 3,460[a]	Unweighted SMD	IPTW[c] Reference group[b]	IPTW[c] Treatment group[b]	IPTW[c] SMD
Sex			0.01			0.02
Female	815 (49.3)	1,716 (49.6)		50.5	49.7	
Residential area			0.24			0.004
North	372 (22.5)	553 (16.0)		18.3	18.2	
Sharon	317 (19.2)	864 (25.0)		22.9	22.9	
South	260 (15.7)	452 (13.1)		13.9	14.0	
Center	295 (17.8)	801 (23.2)		21.4	21.3	
J-lem & Shfela	410 (24.8)	790 (22.8)		23.6	23.6	
Missing	0 (0.0)	0 (0.0)		0.0	0.0	
Socioeconomic status, residential			0.31			0.02
Low	341 (20.6)	480 (13.9)		16.8	16.1	
Medium	642 (38.8)	1,056 (30.5)		33.9	33.8	
High	666 (40.3)	1,918 (55.4)		49.1	49.8	
Missing	5 (0.3)	6 (0.2)		0.3	0.2	
Population subgroup, residential			0.16			0.02
Other/missing	1,458 (88.1)	3,184 (92.0)		90.4	90.9	
Jewish UO	128 (7.7)	216 (6.2)		7.3	6.7	
Arab	68 (4.1)	60 (1.7)		2.3	2.4	
Smoking status			0.09			0.02
Never	1,473 (89.1)	3,156 (91.2)		91.1	90.7	
Ever	177 (10.7)	288 (8.3)		8.5	8.9	
Missing	4 (0.2)	16 (0.5)		0.4	0.4	
BMI category, kg/m^2			0.08			0.02
Normal, 18.5–24.9	351 (21.2)	714 (20.6)		20.5	20.8	
Underweight, <18.5	9 (0.5)	23 (0.7)		0.8	0.6	
Overweight, 25.0–29.9	584 (35.3)	1,286 (37.2)		36.5	36.3	
Obese I, 30.0–39.9	576 (34.8)	1,169 (33.8)		34.6	34.6	
Obese II, 40.0+	58 (3.5)	147 (4.2)		3.8	3.8	
Missing	76 (4.6)	121 (3.5)		3.8	3.8	
Diabetes	624 (37.7)	1,233 (35.6)	0.04	36.2	36.5	–0.01
Cardiovascular disease	573 (34.6)	1,093 (31.6)	0.06	32.5	32.5	0
Hypertension	1,092 (66.0)	2,068 (59.8)	0.13	63.3	62.3	0.02
Chronic kidney disease	718 (43.4)	1,357 (39.2)	0.09	41.0	40.8	0

Characteristic	Unweighted			IPTW[c]		
	Reference group n = 1,654[a]	Treatment group n = 3,460[a]	SMD	Reference group[b]	Treatment group[b]	SMD
Liver disease	423 (25.6)	923 (26.7)	–0.03	25.6	26.1	–0.01
Diseases of the nervous system, prior 12 mo	917 (55.4)	2,010 (58.1)	–0.05	58.5	57.5	0.02
Chronic obstructive pulmonary disease	136 (8.2)	300 (8.7)	–0.02	8.8	8.5	0.01
Cancer, active or treated in prior 5 y	208 (12.6)	511 (14.8)	–0.06	14.5	14.2	0.01
Immunosuppression	465 (28.1)	924 (26.7)	0.03	27.5	27.0	0.01
Hospitalization in prior 180 d	182 (11.0)	353 (10.2)	0.03	10.5	10.5	0
Risk score			0.24			0
n	1,654	3,460		5,022	5,162	
Mean (SD)	5.0 (2.4)	4.5 (2.4)		4.7 (2.4)	4.7 (2.5)	
Median (IQR)	5.0 (3.0, 6.0)	4.0 (3.0, 6.0)		4.0 (3.0, 6.0)	4.0 (3.0, 6.0)	
Range	0.0, 25.0	0.0, 24.0		0.0, 25.0	0.0, 24.0	
Risk score category			0.28			0.04
2	186 (11.2)	685 (19.8)		14.9	17.2	
3	248 (15.0)	645 (18.6)		18.6	16.7	
4+	1,220 (73.8)	2,130 (61.6)		66.5	66.0	
Risk score = 4+	1,220 (73.8)	2,130 (61.6)	0.26	66.5	66.0	0.01
Symptoms questionnaire (between SARS-CoV-2 positive test and index date)			0.29			0
None reported	159 (9.6)	97 (2.8)		5.3	5.3	
1+ reported	833 (50.4)	1,760 (50.9)		50.7	50.6	
Missing	662 (40.0)	1,603 (46.3)		44.0	44.1	
Days since SARS-CoV-2 positive test ≥3	122 (7.4)	157 (4.5)	0.12	5.5	5.3	0.01
COVID-19 vaccination status, n doses			–0.45			0
0	462 (27.9)	365 (10.5)		16.9	17.2	
1	30 (1.8)	61 (1.8)		2.1	1.9	
2	84 (5.1)	183 (5.3)		5.7	5.3	
3	703 (42.5)	1,716 (49.6)		46.0	46.5	
4	375 (22.7)	1,135 (32.8)		29.3	29.1	

Characteristic	Unweighted<br>Reference group<br>n = 1,654[a]	Treatment group<br>n = 3,460[a]	SMD	IPTW[c]<br>Reference group[b]	Treatment group[b]	SMD
Time since last vaccination, if vaccinated, d			0.13			–0.01
n	1,192	3,095		4,174	4,276	
Mean (SD)	131.0 (86.1)	120.1 (85.9)		122.8 (87.3)	124.0 (86.8)	
Median (IQR)	157.0 (39.0, 184.0)	152.0 (32.0, 174.0)		152.0 (33.0, 177.0)	154.0 (35.0, 177.0)	
Range	1.0, 396.0	1.0, 412.0		1.0, 396.0	1.0, 412.0	
Unknown	462	365		848	885	
COVID-19 vaccination status, prior 180 d	850 (51.4)	2,504 (72.4)	–0.44	64.6	64.8	0
Prior SARS-CoV-2 infection	99 (6.0)	128 (3.7)	0.11	4.5	4.7	–0.01

BMI = body mass index; IPTW = inverse probability of treatment weighting; IQR = interquartile range; SARS-CoV-2 = severe acute respiratory syndrome coronavirus 2; SMD = standardized mean difference; UO = ultra-orthodox

[a] All data are n (%) unless otherwise stated.

[b] All data are percentages unless otherwise stated

[c] IPTW was used to adjust for differences in patient characteristics associated with the probability of receiving treatment versus declining treatment. The propensity score model included all characteristics in the table above, as well as "calendar week of index date." Age was included in the propensity score model as a continuous variable (rather than "age group"), and risk score was included as a binary variable ("risk score = 4+", rather than "risk score category"); the SMD associated with these variables was used to assess covariate balance between the two groups.

(B) Patient Characteristics at Index Date, Ages18–64 Years

Characteristic	Unweighted<br>Reference group<br>n = 610[a]	Treatment group<br>n = 1,369[a]	SMD	IPTW[c]<br>Reference group[b]	Treatment group[b]	SMD
Age, y			–0.07			–0.01
Median (IQR)	56.8 (50.4, 61.4)	57.0 (51.7, 61.5)		57.5 (50.6, 61.8)	56.8 (51.4, 61.5)	
≥65	0 (0.0)	0 (0.0)	0	0.0	0.0	0
Sex			0.02			0.04
Female	316 (51.8)	722 (52.7)		53.9	52.0	

Characteristic	Unweighted<br>Reference group<br>n = 610[a]	Unweighted<br>Treatment group<br>n = 1,369[a]	Unweighted<br>SMD	IPTW[c]<br>Reference group[b]	IPTW[c]<br>Treatment group[b]	IPTW[c]<br>SMD
Residential area			0.24			
North	142 (23.3)	217 (15.9)		18.7	18.0	
Sharon	116 (19.0)	348 (25.4)		22.3	23.5	
South	104 (17.0)	208 (15.2)		15.3	15.5	
Center	103 (16.9)	277 (20.2)		21.6	19.6	
J-lem & Shfela	145 (23.8)	319 (23.3)		22.1	23.4	
Missing	0 (0.0)	0 (0.0)		0.0	0.0	
Socioeconomic status (residential)			0.29			0.02
Low	136 (22.3)	189 (13.8)		17.2	16.4	
Medium	229 (37.5)	449 (32.8)		34.8	35.0	
High	244 (40.0)	728 (53.2)		47.8	48.5	
Missing	1 (0.2)	3 (0.2)		0.2	0.2	
Population subgroup (residential)			0.13			0.04
Other/missing	541 (88.7)	1,264 (92.3)		90.6	91.4	
Jewish UO	46 (7.5)	77 (5.6)		7.2	6.2	
Arab	23 (3.8)	28 (2.0)		2.2	2.4	
Smoking status			0.09			0.06
Never	523 (85.7)	1,188 (86.8)		87.0	86.3	
Ever	86 (14.1)	172 (12.6)		12.8	13.2	
Missing	1 (0.2)	9 (0.7)		0.2	0.5	
BMI category, kg/m^2			0.19			0.03
Normal, 18.5–24.9	136 (22.3)	243 (17.8)		19.4	19.1	
Underweight, <18.5	5 (0.8)	9 (0.7)		0.9	0.7	
Overweight, 25.0–29.9	164 (26.9)	415 (30.3)		29.0	28.8	
Obese I, 30.0–39.9	221 (36.2)	534 (39.0)		37.9	38.7	
Obese II, 40.0+	33 (5.4)	97 (7.1)		6.4	6.4	
Missing	51 (8.4)	71 (5.2)		6.4	6.3	
Diabetes	175 (28.7)	416 (30.4)	–0.04	29.3	29.9	–0.01
Cardiovascular disease	104 (17.0)	282 (20.6)	–0.09	18.4	19.2	–0.02
Hypertension	273 (44.8)	585 (42.7)	0.04	44.7	43.6	0.02
Chronic kidney disease	112 (18.4)	327 (23.9)	–0.14	21.2	21.9	–0.02
Liver disease	164 (26.9)	396 (28.9)	–0.05	27.7	28.0	–0.01
Diseases of the nervous system, prior 12 mo	278 (45.6)	704 (51.4)	–0.12	50.5	49.6	0.02

Characteristic	Unweighted Reference group n = 610[a]	Treatment group n = 1,369[a]	SMD	IPTW[c] Reference group[b]	Treatment group[b]	SMD
Chronic obstructive pulmonary disease	19 (3.1)	70 (5.1)	–0.1	4.9	4.4	0.02
Cancer, active or treated in prior 5 y	76 (12.5)	232 (16.9)	–0.13	17.1	15.8	0.04
Immunosuppression	213 (34.9)	466 (34.0)	0.02	36.2	34.5	0.04
Hospitalization in prior 180 d	61 (10.0)	156 (11.4)	–0.05	10.6	10.8	–0.01
Risk score			0.16			0.05
n	610	1,369		1,925	1,995	
Mean (SD)	4.9 (2.1)	4.6 (2.3)		4.8 (2.2)	4.7 (2.3)	
Median (IQR)	5.0 (4.0, 6.0)	4.0 (3.0, 6.0)		4.0 (3.0, 6.0)	4.0 (3.0, 6.0)	
Range	2.0, 17.0	2.0, 23.0		2.0, 17.0	2.0, 23.0	
Risk score category			0.28			0.07
2	48 (7.9)	224 (16.4)		10.2	14.3	
3	96 (15.7)	247 (18.0)		19.8	16.3	
4	466 (76.4)	898 (65.6)		70.0	69.4	
Risk score = 4+	466 (76.4)	898 (65.6)	0.24	70.0	69.4	0.01
Symptoms questionnaire (between SARS-CoV-2 positive test and index date)			0.26			0.03
None reported	46 (7.5)	27 (2.0)		4.2	4.0	
1+ reported	299 (49.0)	716 (52.3)		49.4	51.0	
Missing	265 (43.4)	626 (45.7)		46.4	44.9	
Days since SARS-CoV-2 positive test ≥3	50 (8.2)	82 (6.0)	0.09	6.8	6.7	0.01
COVID-19 vaccination status, n doses			–0.64			–0.02
0	269 (44.1)	217 (15.9)		25.5	25.2	
1	12 (2.0)	37 (2.7)		3.5	2.5	
2	37 (6.1)	105 (7.7)		8.2	7.3	
3	245 (40.2)	793 (57.9)		48.3	51.7	
4	47 (7.7)	217 (15.9)		14.4	13.3	
Time since last vaccination, if vaccinated (days)			0.1			–0.05
n	341	1,152		1,434	1,493	
Mean (SD)	157.2 (79.8)	149.3 (82.8)		147.5 (85.1)	152.0 (83.2)	
Median (IQR)	167.0 (137.0, 183.0)	162.0 (123.5, 180.0)		159.0 (94.4, 181.5)	164.0 (127.3, 181.0)	
Range	2.0, 396.0	2.0, 403.0		2.0, 396.0	2.0, 403.0	
Unknown	269	217		491	502	

Characteristic	Unweighted Reference group n = 610[a]	Unweighted Treatment group n = 1,369[a]	Unweighted SMD	IPTW[c] Reference group[b]	IPTW[c] Treatment group[b]	IPTW[c] SMD
COVID-19 vaccination status, prior 180 d	235 (38.5)	876 (64.0)	–0.53	55.1	55.5	–0.01
Prior SARS-CoV-2 infection	56 (9.2)	77 (5.6)	0.14	6.5	6.7	–0.01

BMI = body mass index; IPTW = inverse probability of treatment weighting; IQR = interquartile range; SARS-CoV-2 = severe acute respiratory syndrome coronavirus 2; SMD = standardized mean difference; UO = ultra-orthodox.

[a] All data are n (%) unless otherwise stated.

[b] All data are percentages unless otherwise stated

[c] IPTW was used to adjust for differences in patient characteristics associated with the probability of receiving treatment versus declining treatment. The propensity score model included all characteristics in the table above, as well as "calendar week of index date." Age was included in the propensity score model as a continuous variable (rather than "age group"), and risk score was included as a binary variable ("risk score = 4+", rather than "risk score category"); the SMD associated with these variables was used to assess covariate balance between the two groups.

(C) Patient Characteristics at Index Date, Ages ≥65 Years

Characteristic	Unweighted Reference group n = 1,044[a]	Unweighted Treatment group n = 2,091[a]	Unweighted SMD	IPTW[c] Reference group[b]	IPTW[c] Treatment group[b]	IPTW[c] SMD
Age, y			0.21			0.02
Median (IQR)	75.6 (71.3, 81.6)	74.3 (69.9, 79.3)		75.2 (70.8, 80.3)	74.7 (70.2, 80.6)	
≥65	1,044 (100.0)	2,091 (100.0)	0	100.0	100.0	0
Sex			0.01			0.01
Female	499 (47.8)	994 (47.5)		48.6	48.3	
Residential area			0.26			
North	230 (22.0)	336 (16.1)		18.3	18.1	
Sharon	201 (19.3)	516 (24.7)		22.9	22.7	
South	156 (14.9)	244 (11.7)		13.1	13.2	
Center	192 (18.4)	524 (25.1)		21.5	22.5	
J-lem & Shfela	265 (25.4)	471 (22.5)		24.2	23.5	
Missing	0 (0.0)	0 (0.0)		0.0	0.0	
Socioeconomic status, residential			0.34			0.02
Low	205 (19.6)	291 (13.9)		16.4	15.8	

	Unweighted			IPTW[c]		
Characteristic	Reference group n = 1,044[a]	Treatment group n = 2,091[a]	SMD	Reference group[b]	Treatment group[b]	SMD
Medium	413 (39.6)	607 (29.0)		33.8	33.2	
High	422 (40.4)	1,190 (56.9)		49.5	50.7	
Missing	4 (0.4)	3 (0.1)		0.3	0.3	
Population subgroup, residential			0.17			0.04
Other/Missing	917 (87.8)	1,920 (91.8)		90.2	90.5	
Jewish UO	82 (7.9)	139 (6.6)		7.4	7.1	
Arab	45 (4.3)	32 (1.5)		2.4	2.3	
Smoking status			0.12			0.06
Never	950 (91.0)	1,968 (94.1)		93.6	93.3	
Ever	91 (8.7)	116 (5.5)		6.0	6.3	
Missing	3 (0.3)	7 (0.3)		0.4	0.3	
BMI category, kg/m^2			0.09			0.03
Normal, 18.5–24.9	215 (20.6)	471 (22.5)		21.7	21.9	
Underweight, <18.5	4 (0.4)	14 (0.7)		0.9	0.6	
Overweight, 25.0–29.9	420 (40.2)	871 (41.7)		40.8	40.7	
Obese I, 30.0–39.9	355 (34.0)	635 (30.4)		32.3	32.3	
Obese II, 40.0+	25 (2.4)	50 (2.4)		2.3	2.3	
Missing	25 (2.4)	50 (2.4)		2.1	2.3	
Diabetes	449 (43.0)	817 (39.1)	0.08	40.1	40.6	–0.01
Cardiovascular disease	469 (44.9)	811 (38.8)	0.12	40.7	40.6	0
Hypertension	819 (78.4)	1,483 (70.9)	0.17	74.5	73.8	0.02
Chronic kidney disease	606 (58.0)	1,030 (49.3)	0.18	52.8	52.6	0
Liver disease	259 (24.8)	527 (25.2)	–0.01	24.3	24.8	–0.01
Diseases of the nervous system (prior 12 mo)	639 (61.2)	1,306 (62.5)	–0.03	63.7	62.4	0.03
Chronic obstructive pulmonary disease	117 (11.2)	230 (11.0)	0.01	11.3	11.1	0.01
Cancer (active or treated in prior 5 y)	132 (12.6)	279 (13.3)	–0.02	13.0	13.2	–0.01
Immunosuppression	252 (24.1)	458 (21.9)	0.05	22.1	22.2	0
Hospitalization in prior 180 d	121 (11.6)	197 (9.4)	0.07	10.3	10.3	0
Risk score			0.28			–0.01
n	1,044	2,091		3,076	3,160	
Mean (SD)	5.1 (2.6)	4.4 (2.5)		4.7 (2.6)	4.7 (2.6)	

	Unweighted			IPTW[c]		
Characteristic	Reference group n = 1,044[a]	Treatment group n = 2,091[a]	SMD	Reference group[b]	Treatment group[b]	SMD
Median (IQR)	5.0 (3.0, 7.0)	4.0 (3.0, 6.0)		4.0 (3.0, 6.0)	4.0 (3.0, 6.0)	
Range	0.0, 25.0	0.0, 24.0		0.0, 25.0	0.0, 24.0	
Risk score category			0.29			0.07
2	138 (13.2)	461 (22.0)		18.4	19.0	
3	152 (14.6)	398 (19.0)		17.8	17.2	
4	754 (72.2)	1,232 (58.9)		63.8	63.8	
Risk score = 4+	754 (72.2)	1,232 (58.9)	0.28	63.8	63.8	0
Symptoms questionnaire (between SARS-CoV-2 positive test and index date)			0.32			0.03
None reported	113 (10.8)	70 (3.3)		6.1	6.1	
1+ reported	534 (51.1)	1,044 (49.9)		50.8	50.4	
Missing	397 (38.0)	977 (46.7)		43.0	43.5	
Days since SARS-CoV-2 positive test ≥3	72 (6.9)	75 (3.6)	0.15	4.7	4.5	0.01
COVID-19 vaccination status, n doses			-0.39			–0.02
0	193 (18.5)	148 (7.1)		11.8	11.9	
1	18 (1.7)	24 (1.1)		1.4	1.4	
2	47 (4.5)	78 (3.7)		4.4	4.1	
3	458 (43.9)	923 (44.1)		44.2	43.4	
4	328 (31.4)	918 (43.9)		38.2	39.2	
Time since last vaccination, if vaccinated, d			0.21			–0.05
n	851	1,943		2,711	2,784	
Mean (SD)	120.5 (86.4)	102.9 (83.0)		110.5 (85.7)	109.0 (85.1)	
Median (IQR)	152.0 (35.0, 184.0)	110.0 (26.0, 169.0)		136.0 (29.0, 174.6)	137.0 (29.0, 173.0)	
Range	1.0, 393.0	1.0, 412.0		1.0, 393.0	1.0, 412.0	
Unknown	193	148		364	376	
COVID-19 vaccination status, prior 180 d	615 (58.9)	1,628 (77.9)	-0.42	70.0	70.7	-0.01
Prior SARS-CoV-2 infection	43 (4.1)	51 (2.4)	0.09	3.2	3.2	0

BMI = body mass index; IPTW = inverse probability of treatment weighting; IQR = interquartile range; SARS-CoV-2 = severe acute respiratory syndrome coronavirus 2; SMD = standardized mean difference; UO = ultra-orthodox

[a] All data are n (%) unless otherwise stated.

[b] All data are percentages unless otherwise stated

[c] IPTW was used to adjust for differences in patient characteristics associated with the probability of receiving treatment versus declining treatment. The propensity score model included all characteristics in the table above, as well as "calendar week of index date." Age was included in the

propensity score model as a continuous variable (rather than "age group"), and risk score was included as a binary variable ("risk score = 4+", rather than "risk score category"); the SMD associated with these variables was used to assess covariate balance between the two groups.

Table S3. HCRU Within 30 Days Post-Index Date, Before and After IPTW, Overall and by Age Group (18–64 Years, ≥65 Years)

(A) HCRU Within 30 Days Post-Index Date, Overall

	Unweighted			IPTW[c]		
**Characteristic**	**Reference group n = 1,654[a]**	**Treatment group n = 3,460[a]**	**SMD**	**Reference group[b]**	**Treatment group[b]**	**SMD**
PCP, ≥1 visit	1,614 (97.6)	3,394 (98.1)	–0.04	97.9	98.2	–0.02
Specialists, ≥1 visit	544 (32.9)	1,263 (36.5)	–0.08	35.8	35.6	0
Telemedicine, ≥1 visit	804 (48.6)	1,931 (55.8)	–0.14	50.3	54.0	–0.07
After-hour urgent care, ≥1 visit	6 (0.4)	20 (0.6)	–0.03	0.3	0.5	–0.03
ER, ≥1 visit	92 (5.6)	203 (5.9)	–0.01	5.10	6.10	–0.04
Hospitalization, all-cause, ≥1 admission	65 (3.9)	97 (2.8)	0.06	3.4	3.1	0.02
Hospitalization. all-cause, LOS			0.23			0.3
n	65	97		168	159	
Median (IQR)	4.0 (2.0, 7.0)	3.0 (2.0, 6.0)		4.0 (2.0, 7.0)	3.0 (2.0, 5.4)	
Hospitalization, all-cause in ICU, ≥1 admission	10 (0.6)	10 (0.3)	0.05	0.5	0.3	0.04
Hospitalization, all-cause in ICU, LOS			1.2			1.1
n	10	10		27	16	
Median (IQR)	15.5 (3.5, 23.5)	2.5 (1.3, 8.0)		11.9 (2.3, 22.7)	3.2 (1.1, 8.0)	
Hospitalization, COVID-19 related, ≥1 admission	34 (2.1)	32 (0.9)	0.09	1.7	1.0	0.06
Hospitalization, COVID-19 related, LOS			0.37			0.56

	Unweighted			IPTW[c]		
Characteristic	Reference group n = 1,654[a]	Treatment group n = 3,460[a]	SMD	Reference group[b]	Treatment group[b]	SMD
n	34	32		87	53	
Median (IQR)	5.5 (3.0, 11.0)	5.0 (3.0, 7.2)		6.0 (3.0, 11.9)	5.0 (2.4, 7.0)	
Hospitalization, COVID-19 related in ICU, LOS			1.5			1.5
n	8	8		22	14	
Median (IQR)	19.0 (12.5, 24.0)	5.5 (1.8, 8.2)		15.5 (6.2, 23.9)	5.0 (1.5, 8.0)	
Maximum level of care			0.1			0.07
None	29 (1.8)	46 (1.3)		1.5	1.3	
Telemedicine	91 (5.5)	203 (5.9)		5.0	5.7	
Outpatient	1,431 (86.5)	3,000 (86.7)		87.7	86.6	
ER	38 (2.3)	114 (3.3)		2.4	3.4	
Inpatient	55 (3.3)	87 (2.5)		2.8	2.8	
ICU	10 (0.6)	10 (0.3)		0.5	0.3	

COVID-19 = coronavirus disease 2019; ER = emergency room; HCRU = healthcare resource use; ICU = intensive care unit; IPTW = inverse probability of treatment weighting; IQR = interquartile range; LOS = length of stay; OR = odds ratio; PCP = primary care physician; SMD = standardized mean difference. *Red: SMD >10%.*

[a] All data are n (%) unless otherwise stated.

[b] All data are percentages unless otherwise stated.

[c] IPTW was used to adjust for differences in patient characteristics associated with the probability of receiving treatment versus declining treatment. The propensity score model included all characteristics in the table above, as well as "calendar week of index date." Age was included in the propensity score model as a continuous variable (rather than "age group"), and risk score was included as a binary variable ("risk score = 4+", rather than "risk score category"); the SMD associated with these variables was used to assess covariate balance between the two groups.

(B) HCRU Within 30 Days Post-Index Date, Ages 18–64 Years

	Unweighted			IPTW[c]		
**Characteristic**	**Reference group, n = 610[a]**	**Treatment group, n = 1,369[a]**	**SMD**	**Reference group[b]**	**Treatment group[b]**	**SMD**
PCP, ≥1 visit	592 (97.0)	1,339 (97.8)	–0.05	97.5	97.9	–0.03
Specialists, ≥1 visit	165 (27.0)	448 (32.7)	–0.12	28.9	31.5	–0.06
Telemedicine, ≥1 visit	347 (56.9)	866 (63.3)	–0.13	57.8	61.1	–0.07
After-hour urgent care, ≥1 visit	2 (0.3)	12 (0.9)	–0.07	0.2	0.8	–0.08
ER, ≥1 visit	33 (5.4)	80 (5.8)	–0.02	5.40	5.80	–0.02
Hospitalization, all-cause, ≥1 admission	18 (3.0)	35 (2.6)	0.02	3.0	2.3	0.04
Hospitalization, all-cause, LOS			0.31			0.4
n	18	35		58	47	
Median (IQR)	3.5 (2.0, 6.8)	4.0 (2.0, 5.5)		5.0 (3.0, 6.7)	4.0 (2.0, 5.4)	
Hospitalization, all-cause in ICU, ≥ admission	4 (0.7)	4 (0.3)	0.05	0.6	0.2	0.06
Hospitalization, all-cause in ICU, LOS			1.3			1.2
n	4	4		12	5	
Median (IQR)	20.0 (12.5, 27.0)	5.5 (2.5, 9.8)		12.6 (2.0, 21.9)	3.5 (1.2, 9.2)	
Hospitalization, COVID-19 related, ≥1 admission	11 (1.8)	13 (0.9)	0.07	2.1	1.0	0.09
Hospitalization, COVID-19 related, LOS			0.41			0.42
n	11	13		40	19	
Median (IQR)	6.0 (3.0, 10.5)	5.0 (3.0, 7.0)		5.4 (3.0, 9.4)	4.0 (3.0, 6.2)	
Hospitalization, COVID-19 related in ICU, LOS			1.2			1.0
n	4	3		12	4	
Median (IQR)	20.0 (12.5, 27.0)	8.0 (5.5, 11.5)		12.6 (2.0, 21.9)	6.1 (3.0, 10.5)	

Characteristic	Unweighted<br>Reference group, n = 610[a]	Treatment group, n = 1,369[a]	SMD	IPTW[c]<br>Reference group[b]	Treatment group[b]	SMD
Maximum level of care			0.09			0.1
None	15 (2.5)	22 (1.6)		2.1	1.6	
Telemedicine	49 (8.0)	104 (7.6)		7.6	7.8	
Outpatient	511 (83.8)	1,159 (84.7)		84.8	84.5	
ER	17 (2.8)	49 (3.6)		2.5	3.7	
Inpatient	14 (2.3)	31 (2.3)		2.4	2.1	
ICU	4 (0.7)	4 (0.3)		0.6	0.2	

COVID-19 = coronavirus disease 2019; ER = emergency room; HCRU = healthcare resource use; ICU = intensive care unit; IPTW = inverse probability of treatment weighting; IQR = interquartile range; LOS = length of stay; OR = odds ratio; PCP = primary care physician; SMD = standardized mean difference.
Red: SMD >10%.

[a] All data are n (%) unless otherwise stated.

[b] All data are percentages unless otherwise stated.

[c] IPTW was used to adjust for differences in patient characteristics associated with the probability of receiving treatment versus declining treatment. The propensity score model included all characteristics in the table above, as well as "calendar week of index date." Age was included in the propensity score model as a continuous variable (rather than "age group"), and risk score was included as a binary variable ("risk score = 4+", rather than "risk score category"); the SMD associated with these variables was used to assess covariate balance between the two groups.

(C) HCRU Within 30 Days Post-Index Date, ≥65 years

Characteristic	Unweighted<br>Reference group n = 1,044[a]	Treatment group n = 2,091[a]	SMD	IPTW[c]<br>Reference group[b]	Treatment group[b]	SMD
PCP, ≥1 visit	1,022 (97.9)	2,055 (98.3)	–0.03	98.3	98.3	0
Specialists, ≥1 visit	379 (36.3)	815 (39.0)	–0.06	39.6	38.4	0.02
Telemedicine, ≥1 visit	457 (43.8)	1,065 (50.9)	–0.14	45.3	49.4	–0.08
After-hour urgent care, ≥1 visit	4 (0.4)	8 (0.4)	0	0.4	0.4	0
ER, ≥1 visit	59 (5.7)	123 (5.9)	–0.01	4.90	6.30	–0.06
Hospitalization. all-cause, ≥1 admission	47 (4.5)	62 (3.0)	0.08	3.7	3.4	0.02

Characteristic	Unweighted<br>Reference group<br>n = 1,044[a]	Unweighted<br>Treatment group<br>n = 2,091[a]	Unweighted<br>SMD	IPTW[c]<br>Reference group[b]	IPTW[c]<br>Treatment group[b]	IPTW[c]<br>SMD
Hospitalization, all-cause, LOS			0.19			0.25
n	47	62		114	108	
Median (IQR)	4.0 (2.0, 7.0)	3.0 (2.0, 5.8)		4.0 (2.0, 7.0)	3.0 (2.0, 6.6)	
Hospitalization, all-cause in ICU, ≥1 admission	6 (0.6)	6 (0.3)	0.04	0.6	0.3	0.03
Hospitalization, all-cause in ICU, LOS			1.2			1.0
n	6	6		17	11	
Median (IQR)	10.0 (3.5, 20.2)	2.0 (1.3, 6.5)		5.3 (2.4, 16.3)	2.0 (1.1, 6.8)	
Hospitalization, COVID-19 related, ≥1 admission	23 (2.2)	19 (0.9)	0.1	1.7	1.0	0.06
Hospitalization, COVID-19 related, LOS			0.35			0.64
n	23	19		53	33	
Median (IQR)	5.0 (2.5, 11.5)	5.0 (2.5, 7.5)		6.4 (2.6, 14.4)	5.0 (2.0, 7.3)	
Hospitalization, COVID-19 related in ICU, LOS			2.1			1.9
n	4	5		11	9	
Median (IQR)	18.5 (12.5, 22.5)	2.0 (1.0, 8.0)		12.4 (5.3, 20.7)	3.3 (1.0, 7.6)	
Maximum level of care			0.11			0.09
None	14 (1.3)	24 (1.1)		1.1	1.2	
Telemedicine	42 (4.0)	99 (4.7)		3.6	4.4	
Outpatient	920 (88.1)	1,841 (88.0)		89.5	87.8	
ER	21 (2.0)	65 (3.1)		2.1	3.2	
Inpatient	41 (3.9)	56 (2.7)		3.2	3.1	
ICU	6 (0.6)	6 (0.3)		0.6	0.3	

COVID-19 = coronavirus disease 2019; ER = emergency room; HCRU = healthcare resource use; ICU = intensive care unit; IPTW = inverse probability of treatment weighting; IQR = interquartile range; LOS = length of stay; OR = odds ratio; PCP = primary care physician; SMD = standardized mean difference

Red: SMD >10%.

[a] All data are n (%) unless otherwise stated.

[b] All data are percentages unless otherwise stated.

[c] IPTW was used to adjust for differences in patient characteristics associated with the probability of receiving treatment versus declining treatment. The propensity score model included all characteristics in the table above, as well as "calendar week of index date." Age was included in the propensity score model as a continuous variable (rather than "age group"), and risk score was included as a binary variable ("risk score = 4+", rather than "risk score category"); the SMD associated with these variables was used to assess covariate balance between the two groups.

Table S4. Odds Ratios of HCRU Within 30 Days Post-Index Date, Before and After IPTW, Overall and by Age Group

	**Overall**				**Age 18–64 years**				**Age ≥65 years**			
	**Unweighted**		**IPTW**[a]		**Unweighted**		**IPTW**[a]		**Unweighted**		**IPTW**[a]	
	**OR**	**95% CI**[a]	**OR**[a]	**95% CI**[a]	**OR**[a]	**95% CI**[a]	**OR**[a]	**95% CI**[a]	**OR**[a]	**95% CI**[a]	**OR**[a]	**95% CI**[a]
PCP, ≥1 visit	1.27	0.85, 1.89	1.16	0.88, 1.54	1.36	0.74, 2.43	1.19	0.78, 1.82	1.23	0.71, 2.08	1.01	0.69, 1.48
Specialists, ≥1 visit	**1.17**	**1.04, 1.33**	0.99	0.91, 1.08	**1.31**	**1.06, 1.62**	1.13	0.99, 1.30	1.12	0.96, 1.31	0.95	0.86, 1.05
Telemedicine, ≥1 visit	**1.34**	**1.19, 1.50**	**1.16**	**1.07, 1.25**	**1.30**	**1.07, 1.58**	**1.14**	**1.01, 1.30**	**1.33**	**1.15, 1.55**	**1.18**	**1.07, 1.30**
After-hour urgent care, ≥1 visit	1.6	0.68, 4.37	1.73	0.94, 3.30	2.69	0.73, 17.3	**3.72**	**1.35, 13.0**	1.00	0.31, 3.75	0.93	0.41, 2.12
ER, ≥1 visit	1.06	0.82, 1.37	**1.2**	**1.02, 1.43**	1.09	0.72, 1.67	1.1	0.84, 1.44	1.04	0.76, 1.45	**1.31**	**1.05, 1.62**
Hospitalization, all-cause, ≥1 admission	**0.71**	**0.51, 0.97**	0.92	0.73, 1.14	0.86	0.49, 1.57	0.77	0.52, 1.13	0.65	0.44, 0.96	0.92	0.70, 1.20
Hospitalization, all-cause in ICU, ≥1 admission	0.48	0.20, 1.16	0.58	0.31, 1.06	0.44	0.10, 1.88	0.38	0.12, 1.03	0.5	0.16, 1.60	0.62	0.28, 1.31
Hospitalization, COVID-19 related, ≥1 admission	**0.44**	**0.27, 0.72**	**0.59**	**0.41, 0.83**	0.52	0.23, 1.20	**0.46**	**0.26, 0.78**	**0.41**	**0.22, 0.75**	**0.59**	**0.38, 0.92**

COVID-19 = coronavirus disease 2019; ER = emergency room; HCRU = healthcare resource use; ICU = intensive care unit; IPTW = inverse probability of treatment weighting; OR = odds ratio; PCP = primary care physician.

Bold: p-value <0.05

[a] IPTW was used to adjust for differences in patient characteristics associated with the probability of receiving treatment versus declining treatment. The propensity score model included all characteristics in the table above, as well as "calendar week of index date." Age was included in the propensity score model as a continuous variable (rather than "age group"), and risk score was included as a binary variable ("risk score = 4+", rather than "risk score category"); the SMD associated with these variables was used to assess covariate balance between the two groups.

Table S5. All-Cause Mortality, Before and After IPTW, Overall and by Age Group

All-Cause Mortality, 30 days post-index: Treatment (n = 3,460) vs. Reference (n = 1,650) group				
	**Unweighted**		**IPTW**[a]	
**Age group, y**	**HR**	**95% CI**	**HR**	**95% CI**
Overall	0.48	0.21, 1.10	0.89	0.50, 1.61
18–64	0.22	0.02, 2.45	0.14	0.02, 1.11
≥65	0.55	0.22, 1.36	0.97	0.51, 1.84

HR = hazard ratio; IPTW = inverse probability of treatment weighting.
Bold: p-value <0.05
[a] IPTW was used to adjust for differences in patient characteristics associated with the probability of receiving treatment versus declining treatment. The propensity score model included all characteristics in the table above, as well as "calendar week of index date." Age was included in the propensity score model as a continuous variable (rather than "age group"), and risk score was included as a binary variable ("risk score = 4+", rather than "risk score category"); the SMD associated with these variables was used to assess covariate balance between the two groups.

Table S6. Odds Ratios of Hospitalization and HCRU Within 30 Days Post-Index Date, Before and After IPTW, for females and males

	**Females**				**Males**			
	**Unweighted**		**IPTW**[a]		**Unweighted**		**IPTW**[a]	
	**OR**	**95% CI**[a]	**OR**[a]	**95% CI**[a]	**OR**[a]	**95% CI**[a]	**OR**[a]	**95% CI**[a]
PCP, ≥1 visit	1.84	0.98, 3.42	**1.66**	**1.07, 2.63**	1.18	0.59, 2.27	0.82	0.50, 1.33
Specialists, ≥1 visit	**1.26**	**1.06, 1.51**	**1.13**	**1.01, 1.27**	1.17	0.95, 1.45	1.02	0.88, 1.17
Telemedicine, ≥1 visit	**1.31**	**1.11, 1.55**	**1.19**	**1.07, 1.33**	**1.40**	**1.14, 1.72**	**1.15**	**1.00, 1.32**
After-hour urgent care, ≥1 visit	1.43	0.42, 6.44	1.73	0.73, 4.41	1.24	0.27, 8.70	1.49	0.43, 5.76
ER, ≥1 visit	1.13	0.78, 1.66	1.33	1.04, 1.71	1.07	0.71, 1.66	**1.39**	**1.04, 1.86**

Hospitalization, all-cause, ≥1 admission	0.79	0.47, 1.33	1.02	0.72, 1.46	0.63	0.38, 1.05	0.84	0.59, 1.18
Hospitalization, all-cause in ICU, ≥1 admission	0.36	0.07, 1.61	0.44	0.15, 1.17	0.50	0.09, 2.68	0.68	0.22, 1.97
Hospitalization, COVID-19 related, ≥1 admission	0.51	0.24, 1.10	**0.54**	**0.32, 0.90**	**0.15**	**0.15, 0.79**	0.65	0.36, 1.16
